# Excited-State Dynamics of Bis(tetraethylammonium) Di-µ-bromo-dibromodicuprate(I) Thin Films

**DOI:** 10.3390/molecules28237841

**Published:** 2023-11-29

**Authors:** Domenic Gust, Mirko Scholz, Kawon Oum, Thomas Lenzer

**Affiliations:** Physical Chemistry 2, Department Chemistry and Biology, Faculty IV: School of Science and Technology, University of Siegen, Adolf-Reichwein-Str. 2, 57076 Siegen, Germany; domenic.gust@uni-siegen.de (D.G.); scholz@chemie.uni-siegen.de (M.S.); lenzer@chemie.uni-siegen.de (T.L.)

**Keywords:** molecular salts, halocuprates, ultrafast laser spectroscopy, triplet emission

## Abstract

Organic–inorganic halocuprates based on monovalent copper are promising luminescent compounds for optoelectronic applications; however, their relaxation processes in the excited electronic state are severely underexplored. In this contribution, we prepare thin films of bis(tetraethylammonium) di-µ-bromo-dibromodicuprate(I) [N(C_2_H_5_)_4_]_2_[Cu_2_Br_4_], abbreviated (TEA)_2_Cu_2_Br_4_, which features a “molecular salt” structure containing discrete [Cu_2_Br_4_]^2−^ anions. This compound, which has an absorption peak at 283 nm, displays a blue, strongly Stokes-shifted emission with a peak at 467 nm. Transient photoluminescence (PL) experiments using broadband emission detection and time-correlated single-photon counting (TCSPC) both find an excited-state lifetime of 57 μs at 296 K. UV–Vis transient absorption experiments at 296 K covering time scales from femto- to microseconds provide evidence for the formation of the T_1_ state through intersystem crossing from S_1_ with a time constant of 184 ps. The triplet state subsequently decays to S_0_ predominantly by phosphorescence. In addition, the time constants for carrier–optical phonon scattering (1.8 ps) and acoustic phonon relaxation (8.3 ns and 465 ns) of (TEA)_2_Cu_2_Br_4_ are provided.

## 1. Introduction

Copper(I)-based materials are promising systems for light-emitting diode (LED) applications because of their high photoluminescence quantum yield and their environmentally benign properties [1,2,3]. One important subgroup are the halocuprates(I), which feature anion chromophores and reach PL quantum yields above 90% [4,5,6,7]. Considerable synthesis efforts have been dedicated to halocuprate(I) systems with closed-shell organic countercations mainly by the groups of Jagner, Willett, and Hartl, and compounds with zero-, one- and two-dimensional anions were obtained [8,9,10]. These early investigations focused on the structural characterization of these compounds without exploring their spectroscopic properties in detail [11].

Only recently, this class of materials has experienced a renaissance, initiated by the drive to find less toxic and more chemically stable alternatives for lead perovskites in LED applications. A range of halocuprates(I) have been explored as crystals, nanoparticles, and thin films featuring inorganic [1,5,6,7,12] or organic countercations [13,14,15,16,17,18]. While these studies found a high PL quantum yield for many of these compounds, a thorough understanding of the underlying luminescence mechanisms is still missing, especially the relation between the photoinduced charge carrier dynamics and the specific structure (0D, 1D, or 2D) of the halocuprate(I) anions and the impact of the countercation.

Detailed spectroscopic information on the electronic relaxation of such compounds can be provided by ultrafast broadband transient absorption and emission experiments. For instance, we recently carried out ultrafast broadband UV–Vis transient absorption measurements on the carrier dynamics of CsCu_2_I_3_ thin films, which feature “infinitely long” [Cu_2_I_3_]*_n_^n^*^−^ anion chains [19]. In these experiments, we detected the disappearance of free exciton (FE) emission as a decay of their stimulated emission band at 320 nm. We were able to provide a time constant for the formation and cooling of self-trapped excitons (STEs) of 12 ps, consistent with a small activation energy in the meV range. The emission band of the STEs was strongly red-shifted and peaked at 564 nm (2.20 eV). These findings were largely confirmed by two later investigations, which suggested additional processes affecting the transient absorption response, such as phonon-assisted cooling and reorganization [20,21].

More recently, we studied the thin films of (CH_3_NH_3_)_4_Cu_2_Br_6_ (denoted as (MA)_4_Cu_2_Br_6_ below), which also showed strongly Stokes-shifted emission with a peak at 530 nm (2.40 eV) [22]. This copper(I) compound represents a 0D “molecular salt” structure with “isolated” [Cu_2_Br_6_]^4−^ ions. Time-resolved PL experiments provided an excited-state lifetime of 114 μs at 296 K. The results from the femto- to microsecond UV−Vis−NIR transient absorption experiments combined with DFT/TDDFT calculations suggested the formation of a long-lived, structurally relaxed triplet species through intersystem crossing (ISC) (61 ps), which almost exclusively decayed by phosphorescence. In addition, time scales for the structural relaxation and cooling processes were extracted from a kinetic analysis of the transient spectra. The calculations for the (MA)_4_Cu_2_Br_6_ crystal and the isolated [Cu_2_Br_6_]^4−^ anion suggested a strong impact of the crystal environment on the structure of the anion [22].

In this paper, we extend our investigations toward another 0D halocuprate(I) system, namely bis(tetraethylammonium) di-µ-bromo-dibromodicuprate(I) ([N(C_2_H_5_)_4_]_2_[Cu_2_Br_4_]), in the following abbreviated as (TEA)_2_Cu_2_Br_4_. This system was originally synthesized and structurally characterized by Asplund and Jagner [23], and its thin-film absorption spectrum was reported by Papavassiliou et al. [24]. More recently, the absorption and emission properties of this compound were investigated in detail by Feng and coworkers [25] and by Liu et al. [26]. Lin, Zeng, and coworkers explored the application of this compound as an X-ray scintillator [27]. There are some conflicting results in the aforementioned studies, for instance, regarding the position of the absorption and emission bands and the excited-state lifetime of this molecular halocuprate(I) salt. In addition, the detailed electronic relaxation processes of (TEA)_2_Cu_2_Br_4_ after photoexcitation are not yet clear. We therefore present here a comprehensive investigation of this compound using steady-state and transient PL spectroscopy and UV–Vis transient absorption measurements in order to clarify the individual relaxation steps in the excited electronic states and the emission mechanism of (TEA)_2_Cu_2_Br_4_ thin films, which were prepared by spin coating.

## 2. Results and Discussion

### 2.1. X-ray Diffraction of (TEA)_2_Cu_2_Br_4_ Thin Films

**Figure 1:**
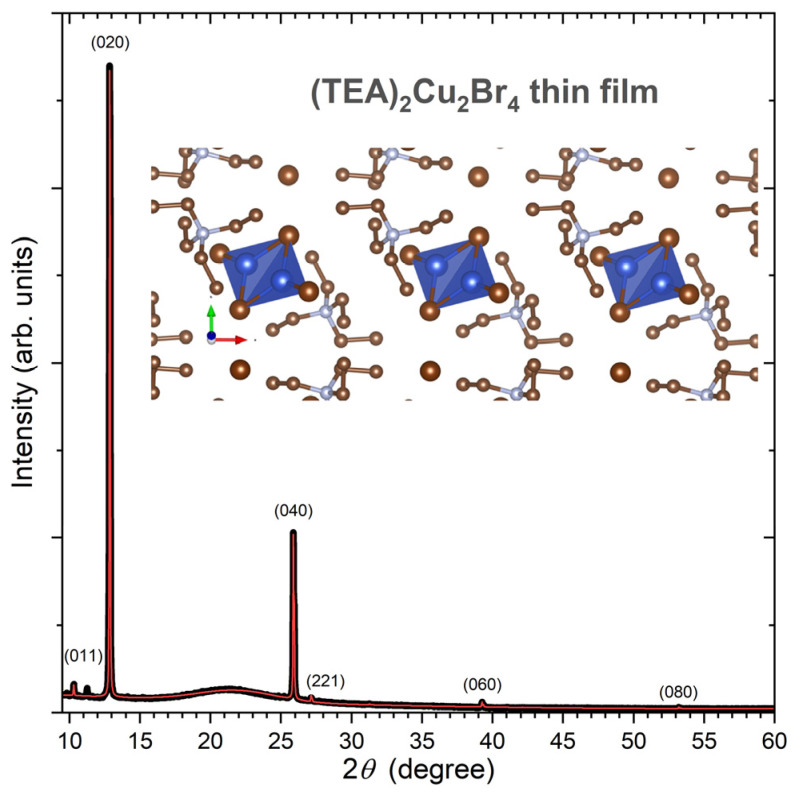
Experimental (black) and simulated (red) XRD pattern of a (TEA)_2_Cu_2_Br_4_ thin film at 293 K. The simulation and *hkl* assignments are based on a Rietveld refinement using the program MAUD [28]. The broad background around 21° arises from the amorphous quartz substrate. On the top right, a zoom-in into the crystal structure of this molecular salt is provided, which contains discrete [Cu_2_Br_4_]^2−^ anions (colors of atoms: Cu (blue), Br (bronze), C (gold), and N (silver)). The structure was visualized using VESTA 3 [29].

Figure 1 shows the experimental X-ray diffraction (XRD) pattern of a (TEA)_2_Cu_2_Br_4_ thin film on quartz (black line). The XRD pattern was analyzed by a Rietveld refinement procedure employing the program MAUD [28], using the structure reported by Chen et al. (monoclinic space group *P* 2_1_/*c*, no. 14) as a starting point [25]. Interestingly, the XRD pattern exhibits a strong texture; therefore, (0*k*0) peaks (*k* = 2, 4, 6, and 8) are dominant. Other reflections, such as (011) and (221), are barely visible. From the fit (red line), we obtained the unit cell parameters *a* = 8.46(5) Å, *b* = 13.7606(4) Å, *c* = 11.08(2) Å, and *β* = 98.3(7)°. These can be, for instance, compared with the values of Chen et al., who found *a* = 8.3505(8) Å, *b* = 13.7641(13) Å, *c* = 11.0507(11) Å, and *β* = 97.076(9)°, based on their single-crystal data [25]. As expected, because of the strong texture effects of the film, only *b* is well defined in our case, and the scatter in the other parameters is expected, because of the limited number of diffraction peaks available. In any case, our XRD pattern confirms the successful synthesis of the (TEA)_2_Cu_2_Br_4_ thin film. A zoom-in into the “molecular salt” structure of this compound featuring isolated [Cu_2_Br_4_]^2−^ anions is provided on the top right of Figure 1. It was obtained using the program VESTA 3 [29]. These anions are discrete centrosymmetric dimers, and the configuration of the bromide ligands around copper(I) is distorted trigonal planar [23].

### 2.2. Steady-State Absorption and Photoluminescence of (TEA)_2_Cu_2_Br_4_ Thin Films

**Figure 2:**
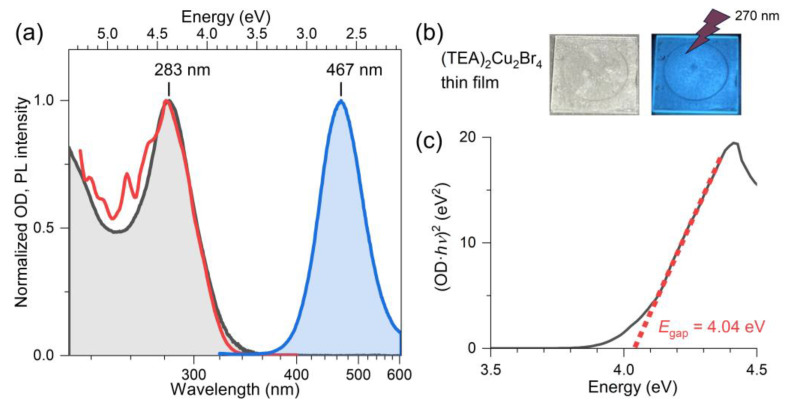
(**a**) Normalized steady-state absorption (black), PL excitation (red, *λ*_em_ = 460 nm), and PL spectra (blue, *λ*_exc_ = 310 nm of a (TEA)_2_Cu_2_Br_4_ thin film on quartz at 296 K. (**b**) Photos of the colorless thin film (**left**) and the blue emission of the film upon photoexcitation with continuous-wave light from a UV–LED with a peak wavelength of 270 nm (**right**). (**c**) Tauc plot of the PL excitation spectrum assuming a direct band gap of the material.

Figure 2a shows the steady-state absorption spectrum (black, baseline-corrected), the PL excitation spectrum recorded at the emission wavelength 460 nm (red), as well as the PL spectrum for the excitation wavelength of 310 nm (blue) for a (TEA)_2_Cu_2_Br_4_ thin film on a quartz substrate. The two photographs in panel b demonstrate that the film is colorless and that illumination by UV light (270 nm) results in an intense blue emission. The absorption spectrum in panel a has a peak at 283 nm (4.38 eV), and its shape is in good agreement with the PL excitation spectrum. The position of the absorption band agrees favorably with previous estimates of Papavassiliou et al. (276 nm) [24] and Liu et al. (280 nm) [26] for this compound. The Tauc plot analysis of the PL excitation spectrum, shown in panel c, which assumes a direct transition (i.e., a linear relationship for (OD·*hv*)^2^ vs. *E* close to the band edge), provides a value of 4.04 eV for the position of the direct band gap (*E*_gap_) of (TEA)_2_Cu_2_Br_4_. The peak of the broad emission band is located at 467 nm (*E*_PL_ = 2.66 eV), and the band has a full width at half maximum (FWHM) of 520 meV. The position of the PL band is in good agreement with the results of Liu et al. (463 nm) [26] and Chen al. (476 nm) [25]. The considerable red shift of the PL band results in a large Stokes shift Δ*E*_Stokes_ = *E*_gap_ − *E*_PL_ of 1.38 eV. The value may be compared with other halocuprates(I), such as CsCu_2_I_3_ (1.57 eV), featuring one-dimensional “infinitely long” [Cu_2_I_3_]*_n_^n^*^−^ chains [19] and the zero-dimensional molecular salt (MA)_4_Cu_2_Br_6_ (1.87 eV) [22]. Characteristic parameters of our spectra of (TEA)_2_Cu_2_Br_4_ (and also kinetic parameters for the transient optical studies reported below) are summarized in Table 1.

### 2.3. Transient PL Spectroscopy of (TEA)_2_Cu_2_Br_4_ Thin Films

**Figure 3:**
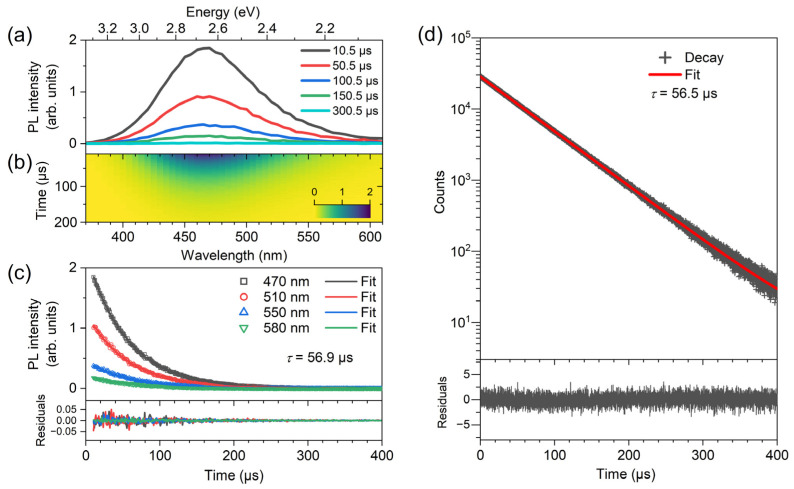
Transient photoluminescence decay of a (TEA)_2_Cu_2_Br_4_ thin film on quartz at 296 K. (**a**) Selected broadband PL spectra at the times indicated. (**b**) Contour map of the PL decay. (**c**) PL kinetics at selected wavelengths after excitation at 310 nm providing a common decay time constant of 56.9 μs. The fit residuals are displayed in the bottom panel. (**d**) Transient PL decay from TCSPC experiments upon excitation by bursts of LED pulses at 273 nm. The TCSPC data are shown as black crosses (one data point every 50 ns), and the red line represents a monoexponential fit with a time constant of 56.5 μs. The fit residuals (dark gray line) are provided in the panel at the bottom.

Figure 3 contains transient PL spectra of a (TEA)_2_Cu_2_Br_4_ thin film, which were recorded over the wavelength range 370–610 nm upon excitation at 310 nm using a combination of a pulsed (80 Hz) xenon lamp and a monochromator. Panel a displays the PL spectra at selected times, and panel b shows the corresponding contour plot. One observes a uniform decay of the PL band. The kinetic traces shown in panel c were analyzed by a monoexponential fit using a shared time constant for all of the decays. The fit provided a value for the time constant *τ*_PL_ of 56.9 μs for the excited-state lifetime of (TEA)_2_Cu_2_Br_4_.

Due to the finite width of the light pulse of the excitation lamp in this setup (FWHM about 2 μs with a substantial tail), PL decays could be recorded only starting from 10 μs. To achieve better coverage of the whole PL decay profile with an improved time resolution and sensitivity, additional transient PL experiments were performed using time-correlated single-photon counting. A bandpass filter at 470 nm (FWHM 10 nm) was employed for detecting the emission of the (TEA)_2_Cu_2_Br_4_ thin film after excitation by bursts of LED pulses at 273 nm. The results of these measurements are shown in panel d of Figure 3. We observed a clean monoexponential decay over almost four orders of magnitude with a time constant *τ*_TCSPC_ of 56.9 μs, in very good agreement with the results of panel c. The value can be compared with previous time constants of 52 μs reported by Liu et al. [26], 58 μs of Lin, Zeng, and coworkers [27], and 53.5 μs provided by Chen et al. [25].

### 2.4. Transient Absorption Spectroscopy of (TEA)_2_Cu_2_Br_4_ Thin Films

**Figure 4:**
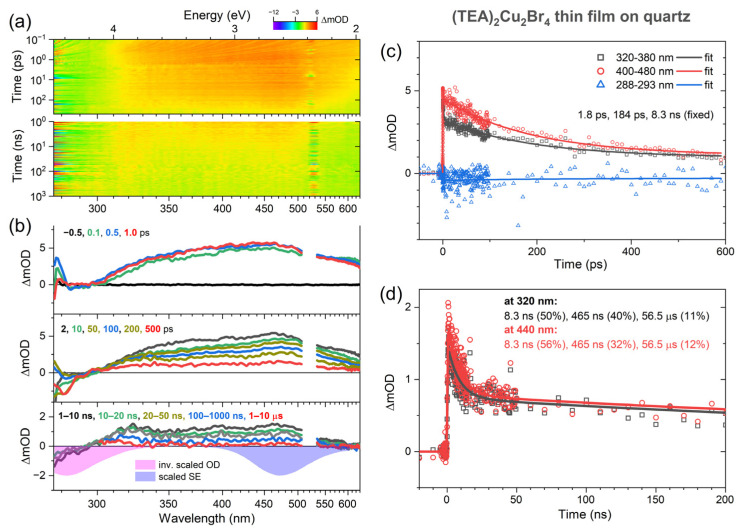
Transient absorption response of a (TEA)_2_Cu_2_Br_4_ thin film on quartz. (**a**) Contour plots for excitation with femtosecond laser pulses at 260 nm (**top**) and nanosecond laser pulses at 266 nm (**bottom**). Increased noise around 520 nm and 532 nm, respectively, arises from the second order of the pump beam stray light in the grating spectrograph. (**b**) Selected spectra from the femtosecond experiments (upper two panels) and the nanosecond measurements (bottom panel) at the time delays indicated. The magenta and violet spectra in the bottom panel correspond to the inverted steady-state absorption spectrum and the steady-state emission spectrum, respectively. (**c**,**d**) Selected kinetics including triexponential fits for the femto- and nanosecond experiments at the wavelengths indicated.

Broadband UV–Vis transient absorption experiments were carried out to investigate the photoinduced dynamics of (TEA)_2_Cu_2_Br_4_ in more detail. They covered a time scale from femtoseconds to microseconds. The thin film was photoexcited with either femtosecond pulses at 260 nm or nanosecond pulses at 266 nm. The results are presented in Figure 4. Panel a contains contour plots of the transient absorption data for the measurements employing femtosecond (top) and nanosecond (bottom) excitation. Panel b shows selected transient absorption spectra at the delay times indicated, and panels c and d contain representative kinetics for time scales up to 600 ps and 200 ns, respectively.

Around zero delay time at the top of panel b, we see the immediate formation of a broad absorption band with a peak at about 470 nm, which spans the entire UV–Vis range. It overlaps with the negative ground-state bleach (GSB) region, which should be located below 330 nm (compare with the magenta-colored inverted steady-state absorption spectrum at the bottom of panel b). The GSB region is also overlapped by stray light contributions of the 260 nm pump pulse (less serious for nanosecond laser excitation at 266 nm). In a localized, “molecular” picture, we assigned the broad absorption band to S_1_ excited-state absorption (ESA) of the “isolated” [Cu_2_Br_4_]^2−^ anions. An initial weak decay of this band with a time constant *τ*_CO_ of 1.8 ps was assigned to vibrational relaxation of the S_1_ state (corresponding to carrier-optical (CO) phonon scattering in a band structure picture of semiconductors).

In the following, the initially formed ESA band decays in an asymmetric fashion. The part around 470 nm decays substantially, whereas there is virtually no change around 320 nm. The resulting change in the band shape is more clearly seen when comparing the spectra in the nanosecond regime (see, for instance, the black spectrum averaged over the time range 1–10 ns), with broad peaks visible around 320 nm and 450 nm. We assigned this process to intersystem crossing from the S_1_ state to the also broadly absorbing T_1_ triplet state, and the time constant *τ*_ISC_ for this decay is 184 ps. We observed similar dynamics in the case of the “molecular salt” (MA)_4_Cu_2_Br_6_, where the ISC process to the T_1_ state was also clearly identified, in that case, with a time constant of 61 ps [22]. The triplet mechanism is also consistent with the absence of a negative stimulated emission (SE) feature, which would be expected to appear around 470 nm (compare with the violet-colored steady-state SE spectrum at the bottom of panel b). Because of the forbidden nature of the T_1_ → S_0_ transition, this SE band must have a very small oscillator strength and is therefore not observable.

**Figure 5:**
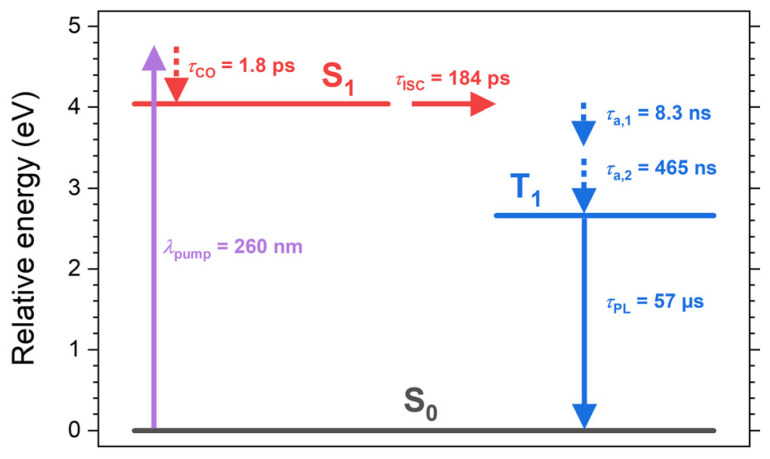
Schematic overview of the electronic states, relaxation processes, and their respective time constants for the thin film of the molecular salt (TEA)_2_Cu_2_Br_4_ on quartz based on the steady-state and time-resolved spectroscopic data summarized in Table 1. *λ*_pump_ indicates an example for an excitation wavelength employed in the femtosecond transient absorption measurements.

On even longer time scales, we see a further decay of the band with the time constants of 8.3 ns and 465 ns. This cannot be the decay from T_1_ to S_0_ (mainly by phosphorescence), because this lifetime was independently determined as 57 μs from the transient PL experiments in Figure 3. Instead, we assigned the dynamics to slower cooling processes in the T_1_ state (corresponding to acoustic phonon relaxation in a band structure picture with the time constants *τ*_a,1_ and *τ*_a,2_), where the substantial excess heat of the (TEA)_2_Cu_2_Br_4_ film is slowly dissipated into the quartz substrate. In fact, quite similar time constants of 12.5 ns and 1.47 μs for the same process were obtained in our previous transient absorption study of (MA)_4_Cu_2_Br_6_ on quartz [22]. The residual very slow decay in the transient absorption signal is consistent with the time constant of 57 μs from the transient PL experiments. Table 1 summarizes the time constants we obtained from our kinetics analysis, and Figure 5 shows a scheme of the electronic states, relaxation processes, and their respective time constants.

Our investigations therefore strongly support a “molecular” phosphorescence process as the emission mechanism of (TEA)_2_Cu_2_Br_4_, similar to what was previously observed for the bromocuprate(I) molecular salt (MA)_4_Cu_2_Br_6_ [22]. This finding is also in line with the results of investigations for related copper(I) systems. For instance, Yersin, Pfitzner, and coworkers observed an almost temperature-independent phosphorescence lifetime of about 180 μs for the compound 1,4-dimethyl-1,4-diazoniabicyclo [2.2.2]octane *catena*-tetra-μ-bromo-dicuprate(I), abbreviated (DABCOMe_2_)Cu_2_Br_4_, over the temperature range 50–200 K, which only slightly decreased to about 80 μs over the temperature range 200–300 K due to a nonradiative quenching mechanism [30]. Boden et al. found a weakly temperature-dependent emission lifetime (0.7–3 μs) with an intermediate maximum for the copper(I) system Cu_4_I_4_(4-Me)_2_ (4-Me = 4-methyl-2-(diphenylphosphino)-pyridine), which was also assigned to a phosphorescence process [31].

These results are somewhat different compared with those for the iodocuprate(I) system CsCu_2_I_3_, which features “infinitely long” [Cu_2_I_3_]*_n_^n^*^−^ chains. In the latter case, the PL was assigned to self-trapped exciton emission [19,32], yet this self-trapped exciton state might indeed also have triplet character. A mechanism based on thermally activated delayed fluorescence (TADF) can be safely excluded, because previous temperature-dependent transient PL experiments for (TEA)_2_Cu_2_Br_4_ by Liu et al. found a weak increase in the excited-state lifetime from about 35 μs at 77 K to 52 μs at 300 K [26]. This is not compatible with a TADF process, because an increase in temperature should make the reverse intersystem crossing (RISC) step from T_1_ to S_1_ faster and should thus lead to a reduction in the lifetime at a higher temperature.

## 3. Materials and Methods

### 3.1. Preparation of (TEA)_2_Cu_2_Br_4_ Thin Films

All preparation steps were carried out under a nitrogen atmosphere. Stoichiometrically equal amounts of CuBr (Sigma-Aldrich, Burlington, MA, USA, 99.999%) and (TEA)_4_NBr (Sigma-Aldrich, 99%) were dissolved in a mixture of dimethyl sulfoxide (Acros Organics, Geel, Belgium, extra dry, 99.7%) and dichloromethane (Fisher Scientific, Waltham, MA, USA, analytical grade, 99.9%) in a 3:2 molar ratio to obtain a solution with a slightly green color, which contained 15 wt% of the nominal composition (TEA)_2_Cu_2_Br_4_. To reduce any residual copper(II) and prevent oxidation of copper(I) to copper(II), two drops of hypophosphorous acid (Alfa Aesar, Ward Hill, MA, USA, 50 wt%) were added, which resulted in a discoloration of the solution. To achieve complete dissolution, the mixture was kept in a screw-cap jar and left for one hour in an ultrasonic bath. Prior to the preparation of the thin films, the quartz substrates (Tempotec Optics Co., Ltd., Fuzhou, China, JGS1) were thoroughly cleaned and then irradiated by UV light for 60 min (Dinies, Villingendorf, Germany, 2 UVC lamps with 11 W each) to remove any residual organic contaminants. Thin films were then deposited by spin coating the “(TEA)_2_Cu_2_Br_4_ solution” onto the substrates at 500 rpm for 30 s and then 2000 rpm for 30 s. Immediately after the spin coating, substrates were post-annealed at 80 °C for 60 min to remove residual solvent. Afterward, a protective layer was spin-coated on top of the halocuprate(I) film (500 rpm for 30 s and then 2000 rpm for 30 s) by using a 12.5 mg mL^–1^ solution of poly(methyl methacrylate) (Alfa Aesar) in anhydrous chlorobenzene (Sigma-Aldrich, 99.8%). The thin films were then annealed at 80 °C for 30 min to remove any solvent residues. The PMMA top layer protected the (TEA)_2_Cu_2_Br_4_ thin films against exposure to oxygen and humidity for short-term studies under atmospheric conditions.

### 3.2. X-ray Diffraction Experiments

The thin-film X-ray diffractograms were recorded on a diffractometer (PANalytical, Almelo, The Netherlands, X’Pert MPD PRO) using Cu radiation (Kα_1_ = 1.54060 Å, Kα_2_ = 1.54443 Å). A Rietveld refinement procedure was applied to simulate the diffractograms using the program MAUD, which included fitting of the baseline and also considered texture effects [28].

### 3.3. Steady-State and Time-Resolved Broadband Photoluminescence Spectroscopy

Steady-state and transient PL measurements of the thin-film samples were performed at 296 K using a spectrophotometer (Agilent, Santa Clara, CA, USA, Cary Eclipse) with the excitation and emission slit widths set at 5 nm. Transient PL decays were recorded over the wavelength range 370–610 nm at a step size of 5 nm after exciting the films at 310 nm using a pulsed xenon lamp (80 Hz, FWHM 2 μs) in combination with a monochromator. To avoid any contamination of the kinetics by contributions of the lamp pulse, the kinetics were collected starting from a delay time of 10 μs. They were recorded up to 1.0 ms, with a gate time of 2 μs and a time resolution of 1 μs. Ten individual kinetics were averaged for each wavelength. The transient spectra were obtained by transposing the set of wavelength-dependent kinetics. Finally, these PL spectra were post-processed by applying a calibration curve, which corrected for the wavelength-dependent sensitivity of the emission monochromator and photomultiplier detector.

### 3.4. Time-Correlated Single-Photon Counting

The TCSPC setup was described in detail previously [33]. Briefly, a UV–LED (Becker & Hickl, Berlin, Germany, UVL-FB-270) with a center wavelength of 273 nm and a pulse width of 500 ps was employed to excite the thin-film samples (*T* = 296 K) at vertical polarization by means of a wire grid polarizer (Thorlabs, Newton, NJ, USA, WP25M-UB). The surface of the thin film was mounted at an angle of 45° with respect to the excitation beam. To record the slow PL decay, the TCSPC module (Becker & Hickl, SPC-130IN, reverse start−stop configuration) was operated in the triggered-accumulation multichannel scaler (MCS) mode. A total of 2 μs long bursts of LED pulses (80 MHz, 160 pulses) were employed at a repetition frequency of 1 kHz. The PL was measured at an angle of 90° after passing a wire grid polarizer (Thorlabs WP25M-UB), which was set at the magic angle of 54.7°, and a bandpass filter with a center wavelength of 470 nm and an FWHM of 10 nm (Thorlabs, FB470-10). Photon detection was performed by a hybrid multialkali photodetector (Becker & Hickl, HPM-100-07) connected to the TCSPC module. The bin width of the recorded decay curve was 50 ns. The time constant of the exponential decay was extracted from a tail fit using the FAST program (Edinburgh Instruments, Livingston, UK).

### 3.5. Femtosecond and Nanosecond Transient Absorption Spectroscopy

Ultrafast broadband transient absorption experiments with up to 1.5 ns delay time were carried out on a setup based on a regeneratively amplified titanium:sapphire system (Coherent, Santa Clara, CA, USA, Libra USP-HE) with a center wavelength of 800 nm and a repetition frequency of 920 Hz. It covers the UV–Vis region (260–700 nm) [34] with a time resolution of about 80 fs and is based on the pump−supercontinuum probe (PSCP) technique [35]. The samples were excited at 460 Hz using the 260 nm output of an OPA system (Coherent, OPerA Solo). In order to access long delay times up to several hundred microseconds, the UV–Vis setup was combined with a Q-switched Nd:YAG microlaser (Standa, Vilnius, Lithuania, Standa-Q1TH), which was externally triggered at 460 Hz and synchronized with the electronics of the ultrafast regenerative amplifier system (jitter less than 350 ps) [22]. The second-harmonic output of the laser was frequency-doubled by an external BBO crystal, and the resulting 266 nm pulses (FWHM ca. 420 ps) were employed for exciting the halocuprate(I) thin film. The film sample (*T* = 296 K) had an optical density of about 0.16 at the excitation wavelength. It was mounted inside a nitrogen-flushed aluminum cell and translated by a piezo stage in the *x*–*y* plane within a sample area of 2 × 2 mm^2^. The initial carrier number density was about 5 × 10^18^ cm^−3^. The kinetics of the charge carrier relaxation was described by a sum of three exponential functions.

## 4. Conclusions

Thin films of the halocuprate(I) (TEA)_2_Cu_2_Br_4_ were investigated by time-resolved emission and absorption spectroscopy from the femto- to microsecond time scales. The luminescence mechanism of this compound is based on the phosphorescence of the long-lived (57 μs) T_1_ triplet state, which is populated from the S_1_ state on a sub-200 ps time scale and is also responsible for the strongly Stokes-shifted photoluminescence. The emission process is governed by the molecular properties of the discrete [Cu_2_Br_4_]^2−^ anions. In contrast, the ammonium-based countercations with aliphatic chains have only high-lying electronic states, which cannot electronically interact with the zero-dimensional halocuprate(I) chromophore. Because of their high photoluminescence quantum yield, halocuprate(I) compounds, such as (TEA)_2_Cu_2_Br_4_, are promising materials for LED applications. First examples of functioning electroluminescent devices have already been demonstrated, yet with quite a small external quantum efficiency below 1% [26]. Beyond optimizing device structures, future work will also need to address the reduction in the width of the emission band in these compounds to achieve a better color purity.

## Figures and Tables

**Table 1:** Summary of the parameters obtained from the steady-state absorption and photoluminescence experiments and time constants extracted from the kinetic analysis of the TCSPC and transient absorption experiments for (TEA)_2_Cu_2_Br_4_ thin films.

Description	Quantity (Unit)	Value
Position of absorption peak	*E*_abs_ (eV)	4.38
Position of direct band gap (from Tauc plot)	*E*_gap_ (eV)	4.04
Position of PL peak	*E*_PL_ (eV)	2.66
Width of PL spectrum	Δ*E*_PL_ (eV)	0.52
Stokes shift (*E*_gap_ − *E*_PL_)	Δ*E*_Stokes_ (eV)	1.38
Carrier-optical phonon scattering	*τ*_CO_ (ps)	1.8
S_1_ → T_1_ intersystem crossing (ISC)	*τ*_ISC_ (ps)	184
Acoustic phonon relaxation	*τ*_a,1_ (ns)	8.3
	*τ*_a,2_ (ns)	465
T_1_ → S_0_ (mainly phosphorescence, ISC negligible)	*τ*_PL_ (μs)	56.9
	*τ*_TCSPC_ (μs)	56.5

## Data Availability

Data are contained within the article.
